# National Board of Health

**Published:** 1882-09

**Authors:** 


### ARTICLE V.

### IMMIGRATION AND DISEASE.

The following report from the Supervising Inspector, and the Secretary of the National Board of Health, is worthy of being placed on record, for the purpose of showing the relations of immigration to the prevalence of diseases of an infectious and contagious character.

The facts stated show clearly enough that our government should enforce more rigidly regulations calculated to prevent the importation of diseases. And if such enforcement should deter some persons from coming, it would be no injury to any legitimate interest on this side of the Atlantic.—ED.

### OPERATIONS IN THE ILLINOIS DISTRICT DURING THE PAST MONTH.

SPRINGFIELD, ILL , AUGUST 4, 1882.

SIR :—I have the honor to submit the following report for the month of July, of the operations of the Immigrant-Inspection Service, N. B. H., in the district under my supervision.

During the month an aggregate of 21,000 immigrants were inspected by the assistant inspectors, who found about sixteen per cent. of these unprotected, liable to contract small-pox and to propagate the disease. The necessary precautions were taken with all of these cases, as well as with one case of varioloid found in transit, and several convalescents, one being an immigrant removed from a train and treated in the small-pox hospital at Rochester during the month of June.

Although there is a marked improvement in the character of the protective work done on shipboard, as compared with that noted on beginning inspections, this is by no means uniform, as will be seen by the following extracts from the assistant inspectors' notes of careless, imperfect or neglected vaccinations and revaccinations :

*Steamship Republic.*—Practically worthless; no adult vaccinations.

*General Herder.*—None showed successful vaccination.

*Strasburg.*—Very poor ; only eleven successful out of 340 vaccinations—result evidently due to want of care on part of steamship surgeon.

*Hermann.*—Only thirteen properly protected out of 410 ; the vaccination performed on shipboard very unsatisfactory. All passengers provided with protection cards.

[These vessels, the *Strasburg* and *Hermann*, are regular Baltimore packets, and the latter, at least, is known to have been the means of introducing small-pox into five localities in Illinois during the past spring.

It might be profitable to inquire to what extent such vessels and the neglect of their surgeons are responsible for the present prevalence of small-pox in Baltimore.]

*Rhynland.*—None showed successful vaccination on shipboard.

*Mosel.*—No successful vaccination by the ship's surgeon.

*Jason* and *Sardinia.*—No ship vaccination met with in passengers from either vessel.

*St. Germain*, *Ethiopia* and *Wieland.*—None vaccinated on shipboard; many totally unvaccinated children provided with ship's protection cards.

*Main* and *Surrey.*—Nearly all unsuccessful.

*Britannic* and *Italy.*—Vaccinations on shipboard generally poor.

*Devonia* and *Warwick.*—Over one-half of the passengers by these two steamers were found to require vaccination, although all were furnished with ship's surgeon's cards, which, they had been told, would prevent any other physician from interfering with them.

*City of Brussels.*—Extremely little attention appears to have been given to the examination of the immigrant passengers while on board. One of these passengers, a Finn, twenty-eight years old, was found on a train nearing Chicago, July 24; was in the eruptive stage—eruption probably 80 hours old. He presented a vaccination-protection card, signed William Gibbons, Surgeon, *SS. City of Brussels*, and dated July 20, 1882.

*Illinois.*—A few only vaccinated.

*Nebraska.*—Poorly revaccinated.

*Indiana.*—Two-thirds required revaccination. No cards had been furnished any immigrants.

Concerning these cards, it is noted that in many instances they bear no name of steamship or line. One such, presented by a lad thirteen years old, and without the slightest evidence of vaccinal protection, bore the name of John W. Watson, Surgeon.

On July 28, among passengers by the steamer *Erin*, were found ten convalescents who had had small-pox within a month before leaving Ireland.

Notwithstanding that the aggregate number of inspections was less in July than in the preceding month, the relative number is much larger. Immigration has probably fallen off one-half since May last, and it is believed that of the total number now arriving, very few find their way into Illinois or cross the State without being subjected to one or more rigorous inspections. The reports of small-pox outbreaks in the region west of the Mississippi river have been notably less during July, while in this State there has not been a single case reported during the month, outside of Chicago. The following table shows the steady decline of the disease in that city:

| Month. | Cases Reported. | Deaths. |
|---|---|---|
| April.................... | 321 | 92 |
| May ..................... | 281 | 65 |
| *June.................... | 124 | 29 |
| July..................... | 44 | 11 |

* Inspection begun June 1.

At the close of the month there were only ten cases under treatment in the city, and one in the State at large.

In other districts of the service it is observed that increased familiarity with the work has secured increased efficiency, and thus materially lightened a labor which was found to be very onerous during the first month. For example: The vaccinations found to be necessary among passengers arriving over the Grand Trunk road in June were very nearly 36 per cent. of the total inspections. During July, owing to the increased number of vaccinations at Port Huron, Mich., this percentage has been reduced to 8.3 per cent.

Many cases of cholera infantum, measles and whooping-cough are met with by the inspectors, who, so far as time and opportunity serve, render such medical assistance as is necessary.

The railway service has continued to afford every desired facility to the inspectors, and, aside from the advent of an occasional unannounced train over a connecting line, every emergency has been promptly met. The coaches for immigrant passengers are kept in good sanitary condition, and the general welfare of these people seems to be consulted on business principles.

Appended is a table showing the number of inspections and of vaccinations by each inspector for the period between June 1 and July 31.

I am, sir, very respectfully,

JOHN H. RAUCH, M.D.,

*Supervising Inspector.*

DR. THOS. J. TURNER, U. S. N.,
*Secretary N. B. H.*,
Washington, D. C.

### IMMIGRANT-INSPECTION SERVICE, N. B. H.

### ILLINOIS DISTRICT.

Inspections and Vaccinations by the Assistant Inspectors: June 1—July 31, 1882.

| INSPECTORS. | STATIONS. | JUNE. | | JULY. | | JUNE 1 to JULY 31 | |
|---|---|---|---|---|---|---|---|
| | | No Inspct'd | No Vacc'd. | No. Inspct'd | No. Vacc'd. | No. Inspct'd | No. Vacc'd. |
| R. E. Starkweather, M.D. | P. Ft. W. & C R | 5895 | 592 | 3452 | 398 | 9347 | 990 |
| Jas. G. Kiernan, M D. | L. S. & M S R R | 4641 | 415 | 1828 | 547 | 6469 | 9[illegible]2 |
| W. F. Bundy, M.D. | M. C. R. R. | 7515 | 1565 | 5835 | 1484 | 13350 | 3049 |
| *J. J. Farrell, M.D. | Gr. Trunk R R | 4028 | 1450 | 2551 | 212 | 6579 | 1662 |
| *F. C. Newton, M.D. | B. & O. R. R. | 2439 | 242 | 2398 | 498 | 4837 | 740 |
| †E. S. Elder, M.D. | Indi napolis. | 992 | 43 | 3079 | 268 | 4071 | 311 |
| W. B. Conery, M.D. | E. St. Louis. | 1511 | 44 | 1784 | 41 | 3295 | 85 |
| Totals. | | 27021 | 4351 | 20927 | 3448 | 47948 | 7799 |

* Inspections began June 1. † Inspections began June 20.

---

